# Cell-free microRNA-21: biomarker for intracranial aneurysm rupture

**DOI:** 10.1186/s41016-020-00195-0

**Published:** 2020-06-12

**Authors:** Hengwei Jin, Yuhua Jiang, Xinke Liu, Xiangyu Meng, Youxiang Li

**Affiliations:** Department of Interventional Neuroradiology, Beijing Neurosurgical Institute and Beijing Tiantan Hospital, Capital Medical University, No.119, South 4th Ring West Road, Fengtai District, Beijing, 100070 China

**Keywords:** Serum miRNA-21, Intracranial aneurysm, Daughter aneurysm, Vascular wall remodeling

## Abstract

**Background:**

Deregulation of miRNA-21 expression has been reported to be associated with vascular smooth muscle behavior and cytoskeletal stability. This study is aimed to investigate the density of serum miRNA-21 in patients with different phases of intracranial aneurysms (IAs) and explore its warning function for IA rupture.

**Methods:**

A total of 16 in 200 IA patients were selected and categorized into 4 groups based on the phase of IA. Microarray study was carried out using serum miRNA and differentially expressed miRNAs were identified. Another 24 samples from a cohort of 360 patients were added and real-time polymerase chain reaction (RT-PCR) was performed on expanded sample size (*n* = 40) for miRNA-21 validation. Potential gene targets of miRNA-21 were screened out from Gene Ontology (GO) database and literatures.

**Results:**

Microarray study identified 77 miRNAs with significantly different expression levels between experimental groups and the control group. RT-PCR assays validated significant downregulation of miRNA-21 in experimental groups, among which miRNA-21 expression level of daughter aneurysm group decreased the most. Bioinformatic analyses revealed that several target genes related with miRNA-21 may be involved in IA formation and rupture.

**Conclusions:**

This study suggested that miRNA-21 had a protective effect for intracranial vascular wall against remodeling and warning function for intracranial aneurysm rupture. Significant suppression of serum miRNA-21 in IA patients may provide diagnostic clues for aneurysm rupture and guide clinical intervention.

## Background

Pathological changes of intracranial aneurysm (IA) include vascular smooth muscle cells (VSMC) apoptosis, matrix degradation, inflammation, and oxidation in the artery wall [1]. However, the biological mechanism of IA process is still unknown. To date, there is no biomarker to monitor the state of imminent rupture, which is the most dangerous state. Daughter aneurysms are focal bulges located at the original IA wall [2]. It is an indicator of the aneurysm rupture. Previous study showed that 57% of ruptured IA had daughter aneurysms, whereas daughter aneurysms were only found in 16% of unruptured IA [3].

MicroRNA (miRNA) is a highly conserved small non-coding single-stranded RNA (20–26 nucleotides in length). It regulates genetic transcription and translation via inhibition or messenger RNA degradation [4]. More than 940 human miRNAs have been recorded [5] and miRNAs have been suggested to regulate about 30% of encoding genes [6] at a post-transcriptional level. One miRNA is capable of targeting several mRNAs and one mRNA may contain several miRNA sites [7]. Phase-specific and tissue-specific are important characteristics of miRNAs. miRNA can resist repeated freeze and thaw cycles and could be tested in serum [8], which makes them attractive biomarkers for various diseases [9].

Deregulation of miRNA-21 expression was reported to be associated with vascular smooth muscle behavior and cytoskeletal stability through regulating tropomyosin- 1[10]. Researchers also revealed that miRNA-21 expressions are different between abdominal aortic aneurysm patients and normal controls [11]. However, no research has proved that miRNA-21 expression level is correlated with IA. In the current study, we investigated the level of serum miRNA-21 in patients with IA of different phases, focusing our attention on aneurysm of high rupture risk. A microarray screening study was followed by validation of miRNA-21 with real-time polymerase chain reaction (RT-PCR). Bioinformatic analyses were carried out to predict target genes of miRNA-21.

## Methods

### Sample acquisition

The study was approved by the Ethics Committee of our institution and performed in accordance with the principles of the Declaration of Helsinki. All the patients signed written informed consent.

**Fig. 1:**
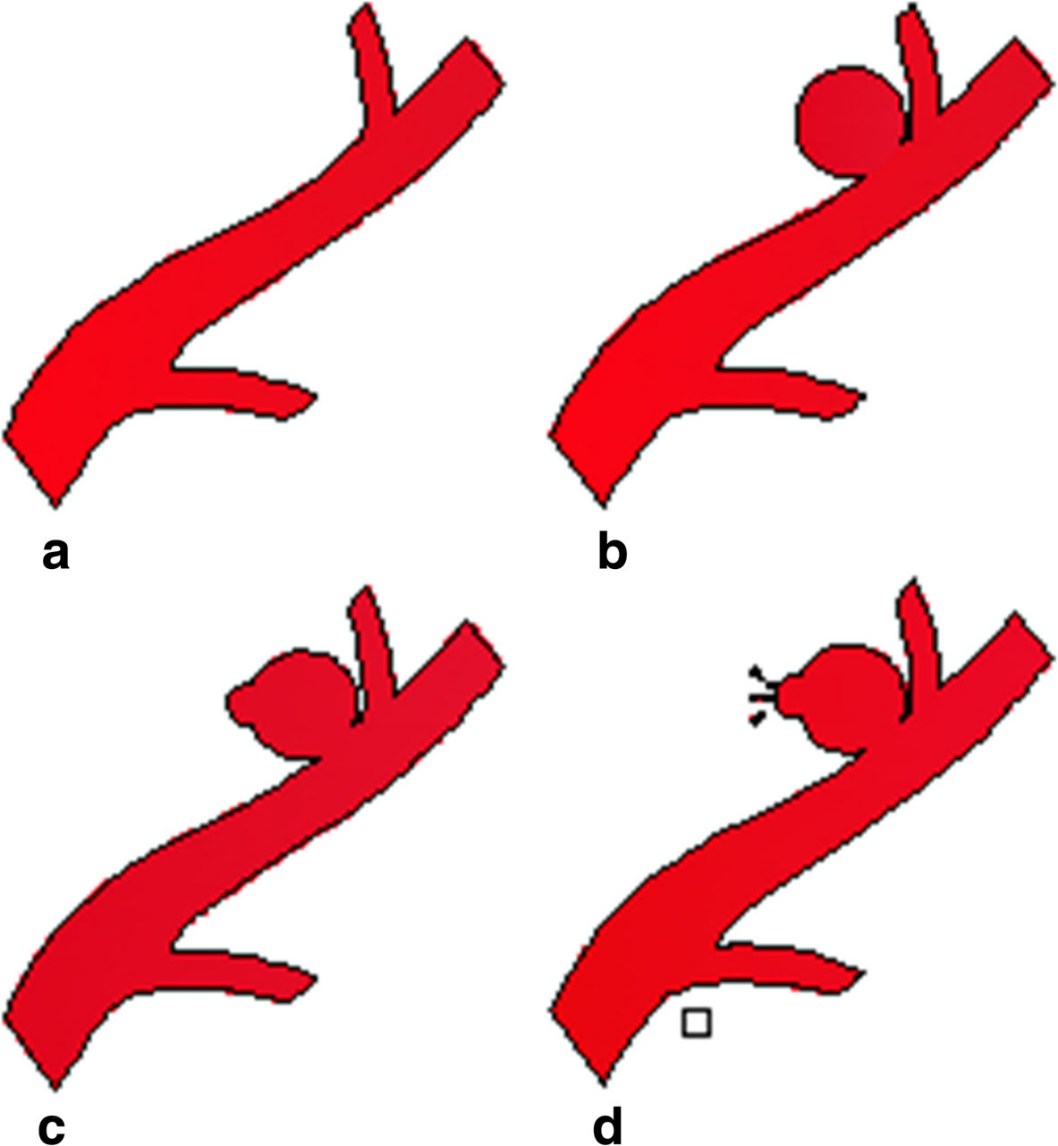
Diagram of 4 groups. **a** Angiography negative group (control group). **b** Aneurysm without daughter aneurysm group (relatively low-rupture risk group). **c** Aneurysm with daughter aneurysm group (relatively high-rupture risk group). **d** Ruptured aneurysm group

In the first step, we selected 16 from 200 IA patients at our department from January to June 2015. All the patients were screening out based on strict criteria (no cerebrovascular disease history such as stenosis and occlusion; no other circulatory system diseases history such as hypertension and cardiac diseases, etc.; no hematologic system disorders, diabetes, and tumor). As was illustrated in Table 1, we divided them into four groups (A-Mi, B-Mi, C-Mi, and D-Mi). A was angiography negative group (control group), B was aneurysm without daughter aneurysm group (relatively low-rupture risk group), C was aneurysm with daughter aneurysm group (relatively high-rupture risk group), and D was ruptured aneurysm group that could be proved by CT (Fig. 1). Microarray study was carried with serum miRNA of these 16 patients.

**Table 1:** General information of all samples

Group	*n*	Gender	Age (years ± SD)
A-Mi	4	2 M, 2 F	42.3 ± 6.9
B-Mi	4	2 M, 2 F	41.2 ± 8.6
C-Mi	4	2 M, 2 F	48.6 ± 9.8
D-Mi	4	2 M, 2 F	46.0 ± 7.5
A-PCR	10	5 M, 5 F	47.3 ± 8.2
B-PCR	10	5 M, 5 F	43.4 ± 7.6
C-PCR	10	5 M, 5 F	43.7 ± 8.1
D-PCR	10	5 M, 5 F	45.4 ± 9.3

*Mi* microarray study, *PCR* real-time polymerase chain reaction, *M* male, *F* female, *A* angiography negative group (control group), *B* aneurysm without daughter aneurysm group (low-rupture risk group), *C* aneurysm with daughter aneurysm group (high-rupture risk group), *D* ruptured aneurysm group that could be proved by CT. Data are mean ± SD

In order to validate the expression difference of miRNA-21, we added the sample size with the other 24 samples selected under the same screening criteria from 360 patients from July to December 2015 (Table 1: A-PCR, B-PCR, C-PCR, and D-PCR). RT-PCR was carried out on the 40 samples. Aneurysms of these patients are located at the internal carotid artery (*n* = 16, 40.0%), posterior communicating artery (*n* = 10, 25.0%), anterior communicating artery (*n* = 9, 22.5%), and posterior circulation (*n* = 5, 12.5%). The size of the aneurysm ranges from 1.0 to 30.0 mm.

### MicroRNA isolation

Blood samples were collected in the morning, no drug interference and intravenous transfusion before operation. Blood samples (2 ml) of the peripheral vein were collected using EDTA tubes. Blood samples were centrifuged at 1200 g for 15 min immediately after collection. Supernatants were transferred into micro-centrifuge tubes. Second centrifugation was performed at 12,000×*g* for 10 min at 48 °C. Serum was collected and stored at − 75 °C for use. Total miRNAs were extracted from serum using mir-VanaTMRNA Isolation Kit (Applied Biosystems, USA). Qualities of miRNAs were assessed using NanoDrop 2000 spectrophotometer. One hundred nanograms of miRNA was taken and added to 2 μm with nuclease-free water for further analysis.

### Microarray study

miRNAs were labeled with miRNA Complete Labeling and Hybridization Kit (Agilent, USA) and hybridized at Human miRNA Microarray Kit (Release 16.0, Agilent, USA), which contained 60,000 probes for 144 human viral and 1205 human miRNAs. Microarray Scanner (Agilent, USA) was used to detect hybridization signals. Feature Extraction Software (Agilent, USA) was used for image analysis. Principal component analysis (PCA) and percentile normalization were performed using Gene spring 12.0. Cluster 3.0 was used for Cluster analysis and differentially expressed miRNAs were screened out.

### MicroRNA-21 expression analysis by quantitative RT-PCR

miRNA-21 was validated by RT-PCR and the small non-coding miRNA-16 was internal control. Primer sequence of miRNA-16 and miRNA-21 were 5′-TAGCAGCACGTAAATATTGGCG-3′ and 5′-TAGCTTATCAGACTGATGTTGA-3′. The reverse transcription was performed for miRNA-21 and miRNA-16 using the miScript II Reverse Transcription Kit (Qiagen, article number 218161). The reverse transcription products were used for the TaqMan real-time PCR reaction with Super Real PreMix (SYBR Green, Tiagen, article number FP205). RT-PCR was performed using the Applied Biosystems 9700 RT-PCR system. The PCR conditions were 95 °C for 15 min, and then 10 s at 95 °C, and 30 s at 60 °C for 40 cycles.

### Bioinformatic analyses

Gene Ontology (GO) database was used to predict genes related to miRNA-21. Network was generated to reveal high-level, summarized views of predicted target genes from the GO database and literatures.

### Statistical analysis

SPSS 18.0 is used for data analysis. The expression difference of miRNAs between groups is analyzed by one-way ANOVA and Dunnett’s multiple comparison test. *P* < 0.05 is regarded as statistical significance.

## Results

### Microarray study

A total of 77 miRNAs are statistically different (*p* < *0.05*) as revealed in Table 2. The expression level of the same miRNA is different among different groups. For example, miRNA-21, miRNA-425, miRNA-27a, miRNA-29a, etc. are downregulated in group C, while upregulated in group B and D. Only a few miRNAs (miR-3679-5p, miR-4271, miR-494, miR-3162, miR-486, miR-92a, miR-765, miR-4298, miR-4327, and miR-940) are upregulated in group C, while upregulated miRNAs account for a large proportion in group B and D. Compared with the control group, the *P* value of miRNA-21 is 0.027 and fold change (FC) of group B, C, and D are 1.51, − 2.31, and 1.25, respectively.

**Table 2:** Differentially expressed miRNAs (*p* < 0.05) screened out in microarray study

	miRNA	*p*	FC (B/A)	*R*	FC (C/A)	*R*	FC (D/A)	*R*
1	hsa-miR-3198	0.000	− 10.41	Down	− 8.81	Down	− 10.18	Down
2	hsa-miR-4314	0.000	− 18.91	Down	− 16.00	Down	− 18.48	Down
3	hsa-miR-425	0.000	1.02	Up	− 59.68	Down	1.07	Up
4	hsa-miR-148b	0.000	2.49	Up	− 7.22	Down	3.21	Up
5	hsa-miR-27a	0.000	1.10	Up	− 149.85	Down	1.23	Up
6	hsa-miR-140-3p	0.000	1.05	Up	− 24.44	Down	− 1.03	Down
7	hsa-miR-101	0.000	2.51	Up	− 15.00	Down	1.77	Up
8	hsa-miR-550a	0.000	− 1.86	Down	− 1.57	Down	− 1.81	Down
9	hsa-miR-148a	0.000	1.74	Up	− 41.09	Down	− 1.24	Down
10	hsa-miR-151-5p	0.000	1.24	Up	− 27.05	Down	1.13	Up
11	hsa-miR-4322	0.000	− 3.53	Down	− 1.98	Down	− 3.44	Down
12	hsa-miR-4286	0.000	− 2.02	Down	− 1.71	Down	− 1.10	Down
13	hsa-miR-3945	0.000	− 7.44	Down	− 6.29	Down	− 7.27	Down
14	hsa-miR-3131	0.000	− 9.32	Down	− 7.89	Down	− 9.10	Down
15	hsa-miR-936	0.000	− 4.34	Down	− 17.62	Down	− 20.36	Down
16	hsa-miR-654-5p	0.000	− 1.74	Down	− 1.48	Down	− 1.70	Down
17	hsa-miR-516b	0.000	− 2.33	Down	− 1.97	Down	− 2.28	Down
18	hsa-miR-139-3p	0.000	− 2.62	Down	− 2.95	Down	− 3.41	Down
19	hsa-miR-4253	0.000	− 4.70	Down	− 9.40	Down	− 10.86	Down
20	hsa-miR-516a-5p	0.000	− 3.65	Down	− 4.08	Down	− 4.71	Down
21	hsa-miR-23b	0.000	1.10	Up	− 17.34	Down	− 1.93	Down
22	hsa-miR-140-5p	0.000	− 2.64	Down	− 44.00	Down	− 1.05	Down
23	hsa-miR-363	0.000	1.54	Up	− 16.54	Down	− 2.20	Down
24	hsa-miR-3679-5p	0.000	1.00	Up	1.91	Up	1.36	Up
25	hsa-miR-19a	0.000	1.21	Up	− 52.68	Down	1.14	Up
26	hsa-miR-342-3p	0.000	− 1.40	Down	− 27.97	Down	− 3.55	Down
27	hsa-miR-199a-5p	0.001	3.64	Up	− 1.89	Down	9.24	Up
28	hsa-miR-27b	0.001	3.27	Up	− 13.00	Down	− 1.10	Down
29	hsa-miR-130b	0.001	3.23	Up	− 5.25	Down	2.75	Up
30	hsa-miR-199a-3p	0.002	− 1.09	Down	− 42.84	Down	1.18	Up
31	hsa-miR-202	0.003	− 2.31	Down	− 28.26	Down	− 1.57	Down
32	hsa-miR-29a	0.004	1.43	Up	− 24.44	Down	1.07	Up
33	hsa-miR-3194	0.005	− 1.95	Down	− 28.97	Down	− 16.38	Down
34	hsa-miR-338-3p	0.005	1.11	Up	− 6.21	Down	2.57	Up
35	hsa-miR-187*	0005	− 2.60	Down	− 7.60	Down	− 8.78	Down
36	hsa-miR-4271	0.005	− 1.35	Down	1.46	Up	− 1.16	Down
37	hsa-miR-548q	0.006	− 5.43	Down	− 22.20	Down	− 16.55	Down
38	hsa-miR-1914*	0.006	− 19.0	Down	− 29.14	Down	− 6.34	Down
39	hsa-miR-221	0.008	1.24	Up	− 10.62	Down	1.40	Up
40	hsa-miR-29b	0.008	4.18	Up	− 2.41	Down	− 1.33	Down
41	hsa-miR-26b	0.008	− 1.09	Down	− 50.92	Down	− 1.58	Down
42	hsa-miR-494	0.008	− 1.79	Down	5.18	Up	− 1.83	Down
43	hsa-miR-3162	0.008	− 1.26	Down	1.39	Up	− 1.00	Down
44	hsa-miR-29c	0.009	2.01	Up	− 17.16	Down	1.63	Up
45	hsa-miR-186	0.009	1.49	Up	− 6.31	Down	− 2.08	Down
46	hsa-miR-107	0.011	1.25	Up	− 21.56	Down	1.07	Up
47	hsa-miR-150	0.011	− 2.59	Down	− 48.16	Down	− 11.70	Down
48	hsa-miR-1226*	0.011	− 5.79	Down	− 18.30	Down	− 6.71	Down
49	hsa-miR-1246	0.013	− 2.39	Down	− 74.43	Down	− 2.40	Down
50	hsa-miR-30b	0014	1.23	Up	− 6.65	Down	1.20	Up
51	hsa-miR-486-5p	0.014	1.28	Up	2.81	Up	1.10	Up
52	hsa-miR-30c	0.015	2.62	Up	− 4.79	Down	− 1.20	Down
53	hsa-miR-92a	0.016	1.29	Up	1.90	Up	1.13	Up
54	hsa-miR-1290	0.017	− 2.82	Down	− 24.13	Down	− 2.55	Down
55	hsv1-miR-H17	0.017	− 1.72	Down	− 2.01	Down	− 1.64	Down
56	hsa-miR-126	0.018	1.39	Up	− 22.40	Down	1.26	Up
57	hsa-miR-185	0.019	1.76	Up	− 12.72	Down	1.77	Up
58	hsa-miR-1268	0.019	− 1.55	Down	− 1.35	Down	− 1.26	Down
59	hsa-miR-765	0.022	− 1.42	Down	1.46	Up	− 1.14	Down
60	hsa-miR-4298	0.022	− 1.41	Down	1.06	Up	1.10	Up
61	hsa-miR-1471	0.022	− 26.60	Down	− 7.22	Down	− 9.89	Down
62	hsa-miR-324-3p	0.024	1.66	Up	− 4.00	Down	− 1.42	Down
63	hsa-miR-130a	0.025	2.40	Up	− 7.23	Down	2.36	Up
64	hsa-miR-21	0.027	1.51	Up	− 2.31	Down	1.25	Up
65	hsa-miR-451	0.027	1.29	Up	− 6.10	Down	1.02	Up
66	hsa-miR-20b	0.028	1.50	Up	− 11.22	Down	− 1.11	Down
67	hsa-miR-2861	0.032	− 1.35	Down	− 1.06	Down	− 1.42	Down
68	hsa-miR-17	0.037	1.56	Up	− 8.64	Down	1.31	Up
69	hsa-miR-4327	0.038	− 2.00	Down	1.41	Up	− 1.51	Down
70	hsa-miR-3937	0.041	− 1.22	Down	− 8.01	Down	− 1.21	Down
71	hsa-miR-1260b	0..046	− 1.50	Down	− 1.68	Down	− 1.95	Down
72	hsa-miR-30e	0.046	2.08	Up	− 7.18	Down	1.41	Up
73	hsa-miR-940	0.046	1.49	Up	4.47	Up	1.18	Up
74	hsa-miR-23a	0.048	− 1.01	Down	− 13.94	Down	− 1.10	Down
75	hsa-miR-484	0.049	− 2.27	Down	− 4.47	Down	− 3.27	Down
76	hsa-miR-19b	0.050	1.34	Up	− 3.20	Down	1.21	Up
77	hsa-miR-1224-5p	0.050	− 5.80	Down	− 11.82	Down	1.13	Up

*p* significant *p* value, *FC* absolute value of fold change, *R* regulation, up means upregulated compared with the control group and down means downregulated. *Low expression. It is a symbol based on naming rule of miRNA. As is revealed, 77 out of all the miRNAs probes have a significantly different expression level

### PCA analysis

**Fig. 2:**
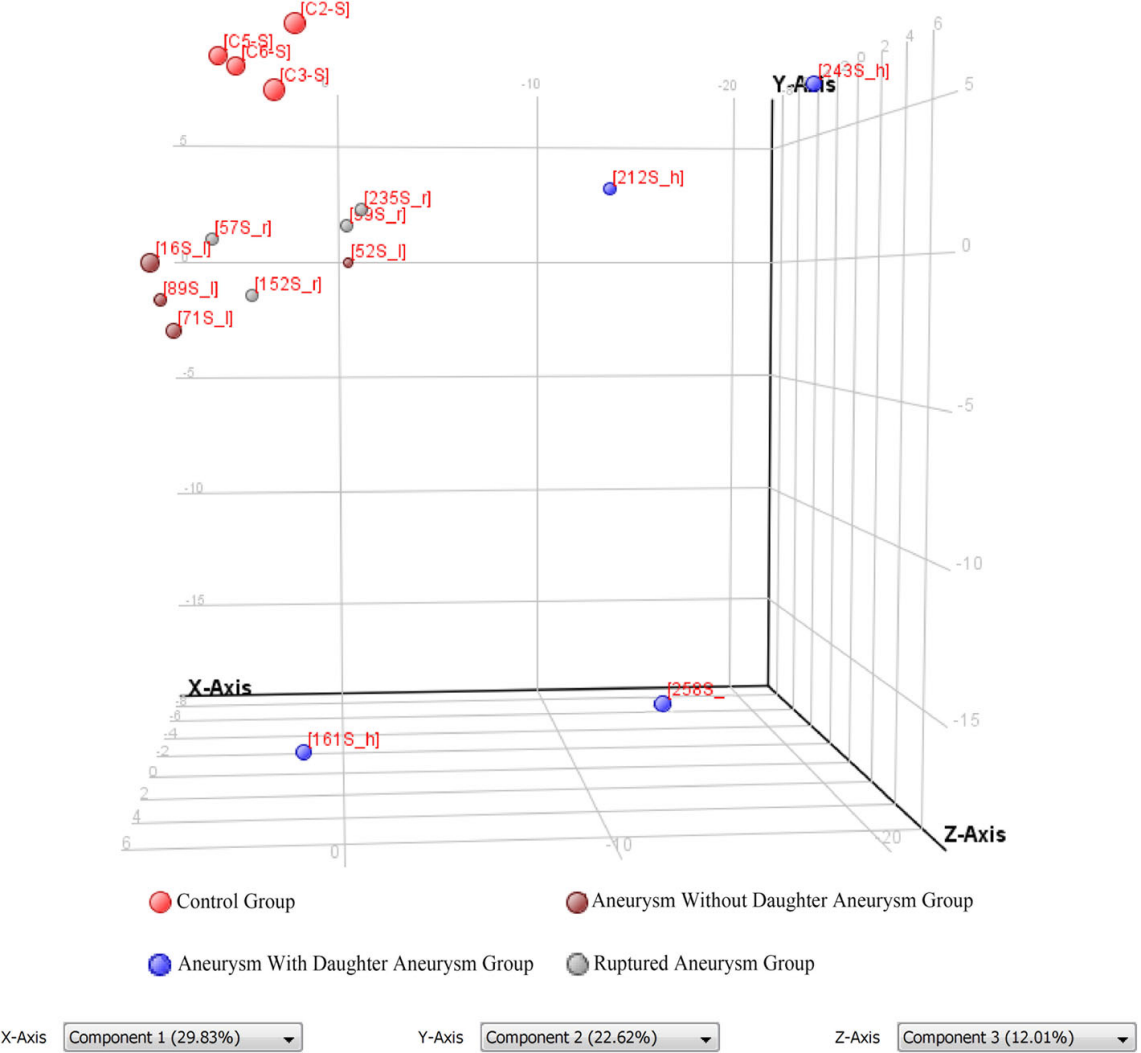
Principal component analysis (PCA). Red, control group; dark red, aneurysm without daughter aneurysm group; blue, aneurysm with daughter aneurysm group; gray, ruptured aneurysm group. The distribution of squares illustrates that miRNAs expression are various among different groups

As illustrated in Fig. 2, patients from the same group are labeled the same color [red, control group (A); dark red, aneurysm without daughter aneurysm group (B); blue, aneurysm with daughter aneurysm group (C); gray, ruptured aneurysm group (D)]. miRNA level varies among groups judged by the distribution of squares, especially between groups A and C.

### Cluster analysis

**Fig. 3:**
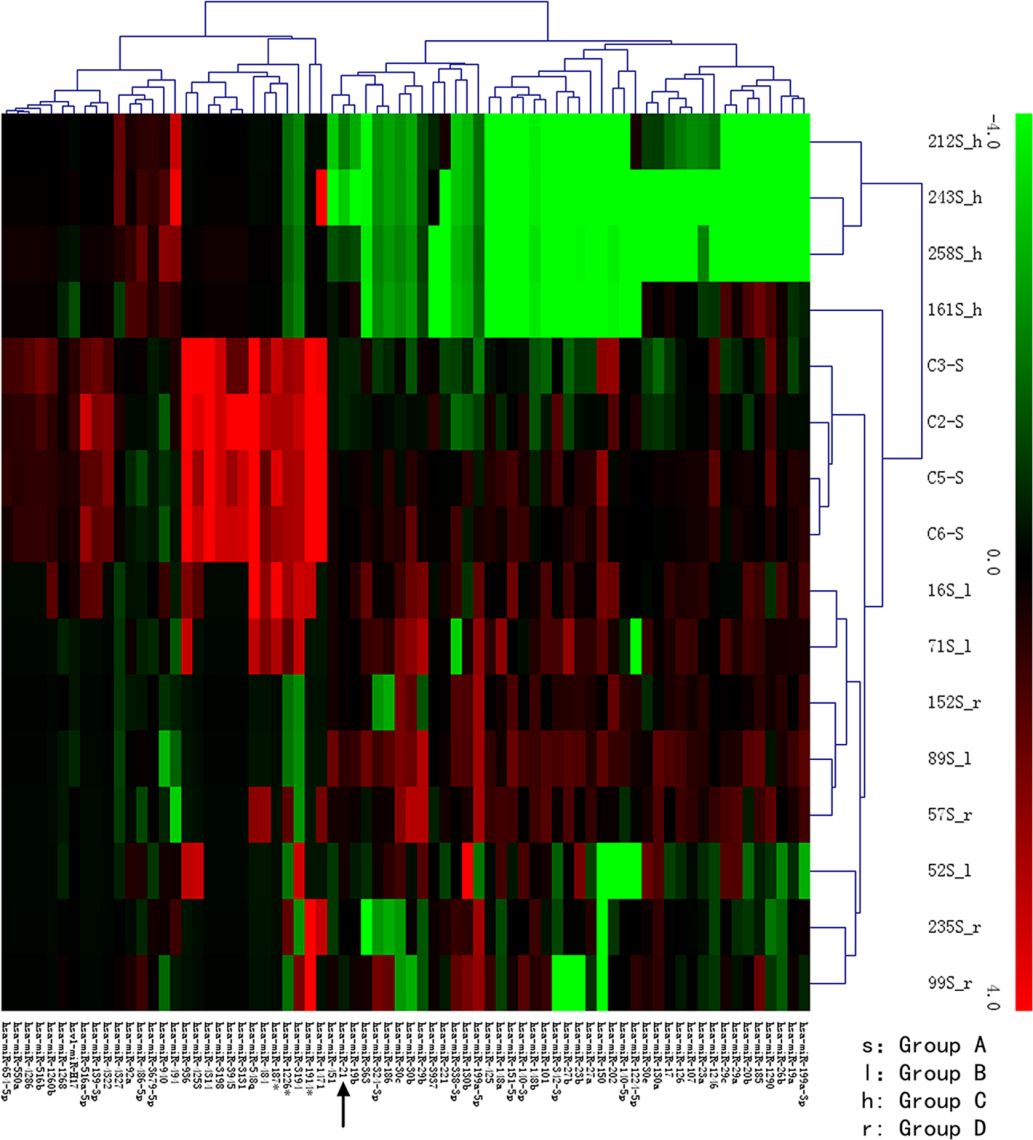
Cluster analysis. C2, C3, C5, and C6 are 4 samples of the control group (A). 16S-l, 71S-l, 89S-l, and 52S-l are low-risk group (B). 212S-h, 243S-h, 258S-h, and 161S-h are high-risk group (C). 152S-r, 57S-r, 235S-r, and 99S-r are ruptured group (D). Green stands for downregulated and red upregulated. Compared with group A, miRNA-21 (dark arrow) of group B and D is upregulated (red), while miRNA-21 of group C is downregulated (green)

C2-S, C3-S, C5-S, and C6-S are 4 samples of group A; 16S-l, 71S-l, 89S-l, and 52S-l are group B; 212S-h, 243S-h, 258S-h, and 161S-h are group C; and 152S-r, 57S-r, 235S-r, and 99S-r are group D (Fig. 3). Green stands for downregulation and red stand for upregulation. miRNAs are differentially expressed among different groups. Compared with group A, miRNA-21(dark arrow) of group B and D is upregulated (red), while group C is downregulated (green).

### MicroRNA-21 expression analysis by quantitative RT-PCR

**Fig. 4:**
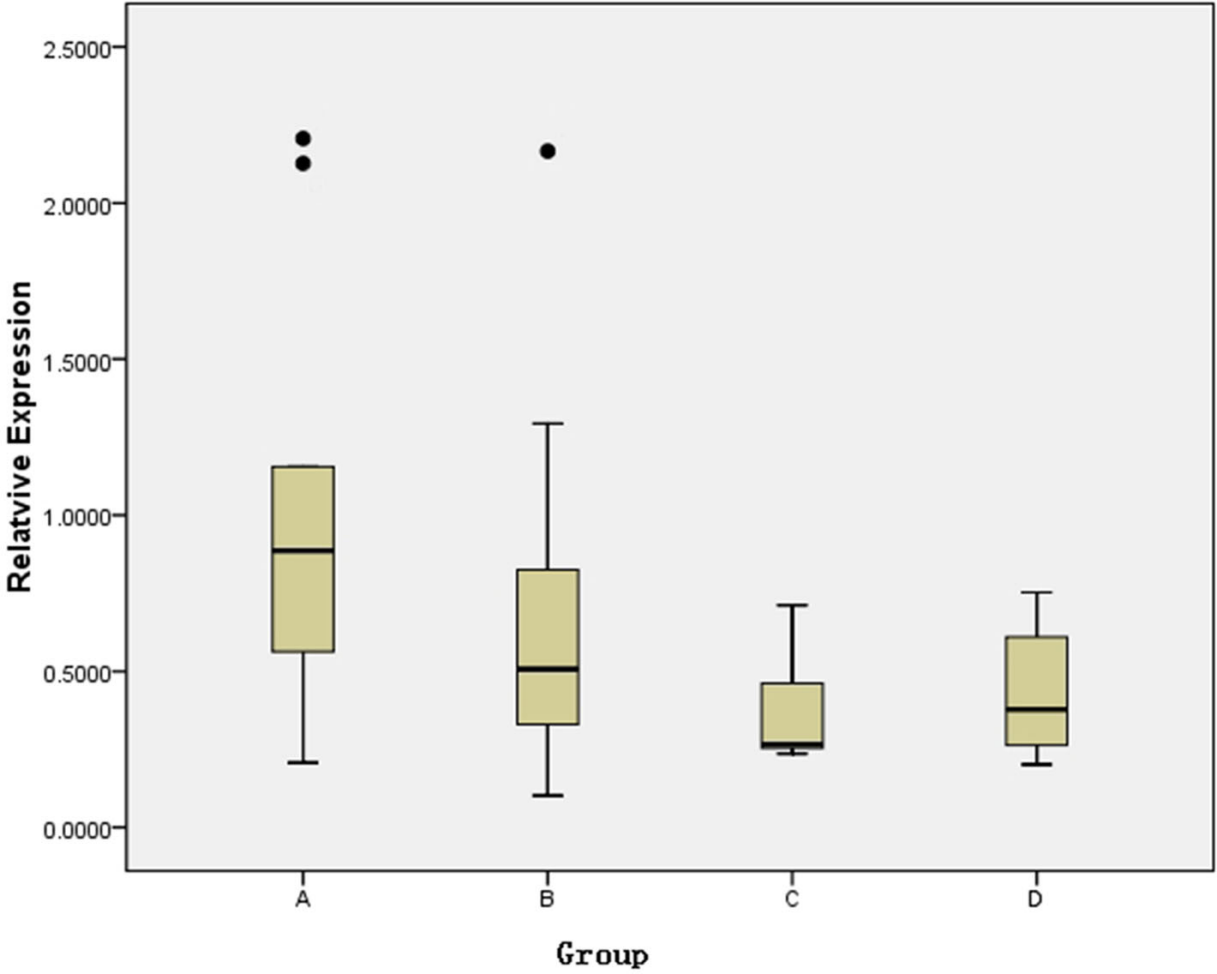
Histogram of qRT-PCR results. *X*-axis represents groups and *Y*-axis represents the relative expression level of miRNA-21. As shown in the figure, compared with the control group, miRNA-21 expression of three experimental groups are downregulated

Relative expression levels of miRNA-21 of 40 samples are listed in Table 3. S1–10 are 10 samples of group A, B, C, and D. Mean expression level of miRNA-21 of four groups are 1.0000, 0.7150, 0.3638, and 0.4372, respectively. *p* value is 0.021. Statistical analysis shows statistical difference between group A and group C (*p* = 0.005), and group A and group D (*p* = 0.013). Histogram demonstrates that the variation tendency of miRNA-21 expression levels among different groups (Fig. 4). *X*-axis means groups A, B, C, and D, and *Y*-axis is relative expression level. Compared with A, the levels of the other 3 groups are all downregulated. No statistical correlation between miRNA-21 level and aneurysm size (*R*^2^ = 0.056) is found.

**Table 3:** Relative expression level of miRNA-21 in RT-PCR

	A	B	C	D
S1	0.2721	2.1670	0.2374	0.2011
S2	0.9586	0.2527	0.2604	0.2205
S3	2.2074	0.4192	0.4619	0.3966
S4	1.1559	0.8249	0.2364	0.6109
S5	0.5983	0.3297	0.5942	0.6025
S6	0.5634	0.1019	0.2545	0.2629
S7	2.1273	1.2945	0.3501	0.7521
S8	0.8136	0.5031	0.2709	0.3413
S9	1.0961	0.7452	0.7115	0.6252
S10	0.2072	0.5113	0.2610	0.3591
Mean expression	1.0000	0.7150	0.3638	0.4372

*S1–S10*10 samples of each group, *A* control group, *B* aneurysm without daughter aneurysm group, *C* aneurysm with daughter aneurysm group, *D* ruptured aneurysm group. *p* value is 0.021. Mean relatively expression levels of four groups are 1.0000, 0.7150, 0.3638, and 0.4372 from A to D, indicating that miRNA-21 of 3 experimental groups are downregulated

### Bioinformatic analyses

**Fig. 5:**
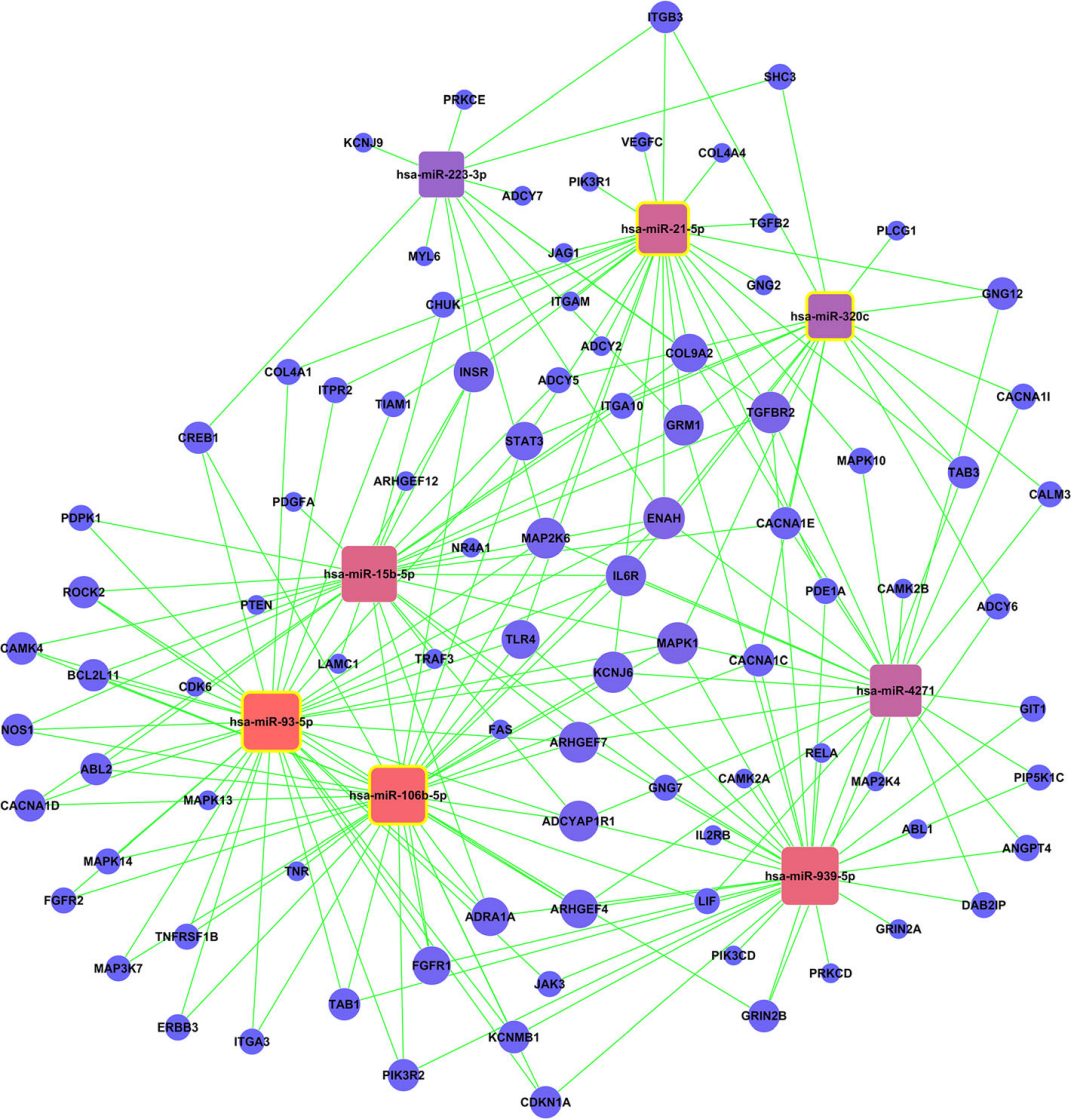
Bioinformatic analysis using Gene Oncology database. As exhibited in the figure, 26 genes and 7 miRNAs are closely related with miRNA-21. Vascular endothelial growth factor C (VEGFC) is directly related with the activity of vascular wall

miRNA-21 target gene analysis was performed using target prediction GO database. A total of 26 genes are closely related with miRNA-21 (Fig. 5). miRNA-223, miRNA-320, miRNA-15b, miRNA-93, miRNA-106b, miRNA-427, and miRNA-939 have some interactions in the regulation of target genes with miRNA-21.

## Discussion

miRNA-21 is one of the most commonly and markedly regulation factor in several cardiovascular disorders and cancers [12]. Expression of miRNA-21 is changed in many vascular diseases. Take the peripheral arterial system as an example, level of miRNA-21 is upregulated following acute vascular injury, and inhibition of miRNA-21 leads to reduced neointima formation [9]. Moreover, miRNA-21 has been verified the function of regulating the activity of vascular muscle cells and signature in human abdominal aortic aneurysms [13]. These results suggest that dynamic changes in miRNA-21 expression level probably occur during aneurysm progression and rupture. However, no study has proved whether miRNA-21 is related with IA. In the effort to probe the relationship between miRNA-21 and IA, we examined the serum miRNA density through microarray study and qRT-PCR. The current study showed that miRNA-21 expression is downregulated in experimental groups and decreases the most in aneurysm with daughter aneurysm group.

Daughter aneurysm is recognized as a high-risk factor of IA rupture [14], which is the most serious outcome of this disease. So daughter aneurysm is separated as an independent group and visualized as an important part of IA development. This is crucial for diagnosis of imminent rupture of aneurysm and guidance of clinical intervention. This mode of grouping will undoubtedly lead us to pay much attention on imminent rupture of IA.

Correlation analysis shows no statistical significance between miRNA-21 expression level and aneurysm size. This agrees with clinical experiments that the rupture risk of aneurysm does not increase with the increase of size. Contradictorily, researchers proved that aneurysms of relatively smaller diameter had a higher risk of rupture than larger aneurysms [15, 16].

It is worth noting that the difference between groups A and C is greater, while the difference between A, and B and D is relatively smaller. This may probably imply that a significant low expression level of miRNA-21 has a warning function for aneurysm rupture. Clustering analysis revealed that compared with group A, the expression of miRNA-21 is upregulated in group B and D (red), while downregulated in group C (green).

The whole progress of aneurysm is a dynamic process. Control group stands for normal expression level of miRNA-21; that of group B is downregulated to a relatively low extent. Then, with the progress of aneurysm development, expression level of miRNA-21 in group C becomes even lower. After the rupture of an aneurysm, the expression of miRNA-21 rises up. Since group C is a relatively high-rupture risk group and miRNA-21 expression of this group is extremely low, the hypothesis could be developed that miRNA-21 could have a protective effect for vascular wall and warning function for aneurysm rupture. When the body received the impact of internal or external stimulus, miRNA-21 expression level becomes downregulated. As a result, its protection for vascular wall becomes weaker and aneurysm forms as a consequence of vascular remolding. If the stimulus lasts long enough, miRNA-21 expression will continue to fall, and daughter aneurysm formation and aneurysm rupture risk will increase. Expression of miRNA-21 rising up again after rupture may be because of some protective feedback mechanism activation in the body or removal of stimulus. The biggest suppression of miRNA-21 in group C deserves our attention because this may provide diagnostic and therapeutic clues for aneurysm rupture.

During the whole process of this rigorous study, the result of the first part (microarray) is not totally consistent with that of the second part (RT-PCR). Compared with the control group, microRNA-21 expression of groups B and D is upregulated in microarray study, while that of RT-PCR is downregulated. This may result in selection bias or small sample size. Since quantitative method (PCR) is more accurate than qualitative method (microarray). And sample size is bigger in PCR. So the authors are more inclined to the result of RT-PCR.

**Fig. 6:**
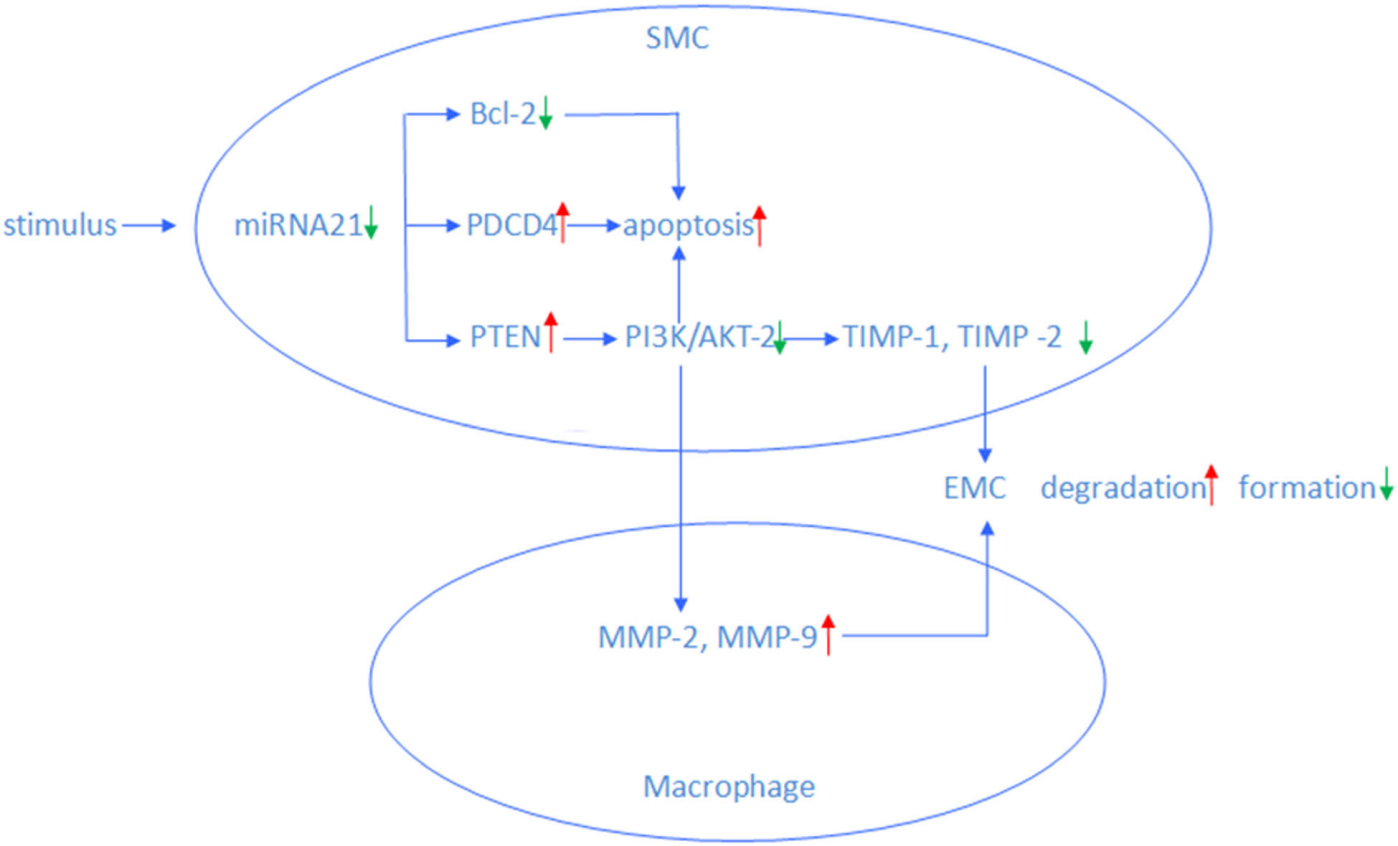
Schematic of miRNA-21 regulation of vascular activity. PTEN, phosphatase and tensin homolog protein; SMC, vascular smooth muscle cell; PDCD4, programmed cell death 4; Bcl-2, B cell lymphoma 2; AKT, serine/threonine protein kinase; Spry1, sprouty homologue 1; MMPs, matrix metalloproteinase. TIMPs, Tissue Inhibitor of Metalloproteinase. As is revealed, downregulation of miRNA-21 resulted from stimulus may lead to increase of SMC apoptosis, decrease of EMC formation, and increase of degradation

In all, 26 target genes that are closely related with miRNA-21 were screened out using the GO database. Vascular endothelial growth factor C (VEGFC) is directly related with the activity of vascular wall from GO analysis. In addition, miRNA-21 may regulate vascular smooth muscle activity via other targets such as phosphatase and tensin homolog protein (PTEN), Bcl-2, and PDCD-4 (Fig. 6). Low expression of miRNA-21 increased the expression of PTEN, leading to decreased phosphorylation and activation of AKT, a part of an anti-apoptotic and pro-proliferative pathway to limit IA expansion and vascular disorder progress [17]. Programmed cell death 4 (PDCD4) is a target gene of microRNA-21. In the serum deprivation apoptotic model, overexpression of PDCD4 increases vascular smooth muscle cell (VSMC) apoptosis. In contrast, VSMC apoptosis is obviously decreased by knock-down of PDCD4 [18]. Activator protein 1 (AP-1) is a downstream molecule of PDCD4 that is associated with VSMC apoptosis. miRNA-21 inhibition decreases and overexpression increases B cell lymphoma 2 (Bcl-2) expressions [9]. Although the molecular mechanism is unclear, the results suggest that low expression of Bcl-2 mediated by miRNA-21 leads to increase of vascular muscle cell apoptosis. Moreover, Wang et al. articulated that miRNA-21 was located in arterial smooth muscle cell (ASMC) and regulated VSMC migration and proliferation migration via targeting tropomyosin 1 (TPM1) [13]. This implies complex regulation of miRNA in the VSMC compartment in reaction to pathological stimulation. miRNA-21 expression is increased in fibroblasts of heart failure, activating ERK-MAP kinase through inhibition of sprouty homologue 1 (Spry1). Fibroblast survival and growth factor secretion are regulated through this mechanism [19].

In addition to ASMC apoptosis, decreased formation and/or increased degradation of extracellular matrix (ECM) are also believed to be key pathophysiological processes leading to aneurysm formation and rupture [20]. The process of IA development involves dysregulation of ECM synthesis, deposition, and remodeling. The role of ECM dysregulation in IA formation is acknowledged; the upstream signaling pathways that initiate the process are still unknown. Studies showed that enhanced matrix metalloproteinase (MMPs) production promotes abdominal aneurysm formation by increasing ECM destruction. MMPs are mainly secreted by leukocytes. MMP-2 and -9 are involved in proteolytic degradation of the ECM. MMP-2 can degrade both collagen and elastin [21], while Tissue Inhibitor of Metalloproteinase (TIMPs) regulates the activity of MMPs by forming complexes with them. It is likely that an imbalance between MMP and TIMP leads to ECM formation decrease and degradation increase. This leads to aneurysm formation, progression, and rupture [22]. Previous studies have proved that miRNA-21 protects vascular via inhibition of vascular wall remodeling. This is consistent with the result of this study. When the expression of miRNA-21 decreases, its protective efficiency declines and an aneurysm is formed as a result of wall remodeling. The lower the expression level of miRNA-21 is, the higher the risk of daughter aneurysm formation and aneurysm rupture will be. Even though the specific quantity remains unknown, this study shows warning function for IA rupture.

The study had some limitations. There is some bias because the sample size is small. For example, the result of microarray study is not coinciding exactly with that of RT-PCR. The classification of the high-rupture risk group is lacking specific criteria. In addition, there are some limitations using serum miRNA as biomarkers. Moreover, the result and hypothesis of this study were not validated with protein analysis. Much effort is needed before the protective effect and warning function of miRNA-21 could be used in diagnostic and therapeutic applications in IA rupture.

## Conclusions

This study suggested that miRNA-21 may have had a protective effect for intracranial vascular wall against remodeling and warning function for intracranial aneurysm rupture. Significant suppression of serum miRNA-21 in IA patients may provide diagnostic clues for aneurysm rupture and guide clinical intervention.

## Data Availability

All data generated or analyzed during this study are included in this published article.

## Abbreviations

IA: Intracranial aneurysm
miRNA: microRNA
qRT-PCR: Quantitative real-time polymerase chain reaction
VSMC: Vascular smooth muscle cells
PCA: Principal component analysis
PTEN: Phosphatase and tensin homolog protein
ECM: Extracellular matrix
